# The Contribution of Epigenetics to Evolutionary Adaptation in *Zingiber kawagoii* Hayata (Zingiberaceae) Endemic to Taiwan

**DOI:** 10.3390/plants12071558

**Published:** 2023-04-04

**Authors:** Yi-Shao Li, Pei-Chun Liao, Chung-Te Chang, Shih-Ying Hwang

**Affiliations:** 1 School of Life Science, National Taiwan Normal University, 88 Tingchow Road, Section 4, Taipei 11677, Taiwan; 2 Department of Life Science, Tunghai University, 1727 Taiwan Boulevard, Section 4, Taichung 40704, Taiwan; changchuante@gmail.com

**Keywords:** environmentally associated epigenetic variation, isolation-by-distance, isolation-by-environment, local adaptation, redundancy analysis, *Zingiber kawagoii*

## Abstract

We epigenotyped 211 individuals from 17 *Zingiber kawagoii* populations using methylation-sensitive amplification polymorphism (MSAP) and investigated the associations of methylated (mMSAP) and unmethylated (uMSAP) loci with 16 environmental variables. Data regarding genetic variation based on amplified fragment length polymorphism (AFLP) were obtained from an earlier study. We found a significant positive correlation between genetic and epigenetic variation. Significantly higher mean mMSAP and uMSAP *uH*_E_ (unbiased expected heterozygosity: 0.223 and 0.131, respectively, *p* < 0.001) per locus than that estimated based on AFLP (*uH*_E_ = 0.104) were found. Genome scans detected 10 mMSAP and 9 uMSAP *F*_ST_ outliers associated with various environmental variables. A significant linear fit for 11 and 12 environmental variables with outlier mMSAP and uMSAP ordination, respectively, generated using full model redundancy analysis (RDA) was found. When conditioned on geography, partial RDA revealed that five and six environmental variables, respectively, were the most important variables influencing outlier mMSAP and uMSAP variation. We found higher genetic (average *F*_ST_ = 0.298) than epigenetic (mMSAP and uMSAP average *F*_ST_ = 0.044 and 0.106, respectively) differentiation and higher genetic isolation-by-distance (IBD) than epigenetic IBD. Strong epigenetic isolation-by-environment (IBE) was found, particularly based on the outlier data, controlling either for geography (mMSAP and uMSAP *β*_E_ = 0.128 and 0.132, respectively, *p* = 0.001) or for genetic structure (mMSAP and uMSAP *β*_E_ = 0.105 and 0.136, respectively, *p* = 0.001). Our results suggest that epigenetic variants can be substrates for natural selection linked to environmental variables and complement genetic changes in the adaptive evolution of *Z. kawagoii* populations.

## 1. Introduction

Adaptation to changing environments is a fundamental process for the survival of populations and species, especially during fast-paced environmental changes [1,2,3,4]. Epigenetics, which can occur without alterations in DNA sequences, is an important mechanism influencing population processes [5]. Over the ecological and evolutionary time scales of range redistribution, mutation and recombination would rarely provide sufficient sources of variation [6]. Epigenetic modifications have been suggested to respond to the environment before genetic changes begin to accumulate [7]. Phenotypic plasticity may arise as a result of epigenetic switches to deal with fluctuating environments [8], which may buy time for populations in the initial stages of adaptation [9,10]. Epigenetic modifications can generate heritable phenotypic variation in relation to adaptive evolution [11,12]. Population adaptive divergence in association with environmental gradients cannot be explained solely by DNA sequence variation [6]. Stable heritable epialleles may have significant evolutionary roles and are ecologically relevant in natural populations [13,14,15,16].

Epigenetic variation can be classified into (1) obligatory dependence of epigenetic variation on genetic variation, (2) facilitated epigenetic variation represents partial independence of epigenetic variation from genetic variation, and (3) pure epigenetic variation characterizes complete independence of epigenetic variation from genetic variation [17]. In natural plant populations, epigenetic variation can influence evolutionary processes in ways that are not related to sequence variation when epigenetic variation has arisen independently of genetic variation [6,17]. However, epigenetic variation may be the direct or indirect consequence of upstream genetic changes [6,16,17,18]. Epigenetic variation, provoked by DNA methylation and demethylation, is an additional system compensating for adaptive genetic divergence [7,9,10]. Gene flow between populations may be reduced due to isolation-by-distance (IBD) [19]. IBD is the process by which geographically restricted gene flow generates a genetic structure indicating a positive correlation between genetic differentiation and geographic distance. The spatial genetic and epigenetic structure may differ, causing the difference between genetic and epigenetic IBD [20]. Additionally, ecological factors in divergent environments may lead to the selection-driven divergence that decreases gene flow between populations, creating a pattern of isolation-by-environment (IBE) [21,22].

In an earlier study, using data on amplified fragment length polymorphism (AFLP), a latitudinal pattern of environmentally dependent adaptive genetic divergence was found to be highly correlated with the annual temperature range in *Z. kawagoii* [23]. It is probable that the latitudinal northerly range expansion of *Z. kawagoii* [24] may have been related to the latitudinal pattern of adaptive divergence in *Z. kawagoii* [23]. Additionally, the lack of genetic variation in *Z. kawagoii* [23] may result in genetic as well as migration load [25,26]. Studies have demonstrated local adaptation linked to epigenetic variation and closely associated with environmental gradients in a variety of natural systems [27,28,29,30,31,32,33,34]. Therefore, the investigation of epigenetic variation in association with specific environmental variables is important. Epigenetic variation may play a role in compensation for the lack of genetic variation [7,9,10]. Testing for the association between epigenetic variation and environmental factors within a species’ distribution range is important to identify environmental variables that may act as ecological drivers shaping the spatial epigenetic structuring of natural plant populations [6,16,27,30,31,32,33,34]. The association between epigenetic variation and the environment may be related to alteration in the methylation status of different genes, resulting in local adaptation related to fitness-related traits [28,34,35,36].

Epigenetic variation can be quantified with methylation-sensitive amplification polymorphism (MSAP) [37] that reflects a modification in cytosine methylation states [6,27,36]. In the present study, we surveyed the epigenetic variation in 211 individuals from 17 populations using MSAP to test the role that the environment plays in shaping adaptive epigenetic variation in *Z. kawagoii*. Epigenetic variation in association with environmental gradients can be important for plant adaptations to various environments apart from adaptive genetic divergence [5,6,7,9,10,11,12,13,14,15,16]. In addition to previous studies [13,14,15,16,27,28,29,30,31,32,33,34], the present study can provide additional empirical evidence for adaptive epigenetic divergence in natural plant populations. We aimed to answer questions related to spatial epigenetic structuring and environmentally dependent adaptive epigenetic divergence in *Z. kawagoii*. First, we explored the inter-population correlation between epigenetic distance and genetic distance using the Mantel test. Second, we intended to understand the level of population epigenetic diversity and epigenetic structure relative to the level of genetic diversity and genetic structure. Third, we assessed the correlations between all MSAP loci and environmental variables using a latent factor mixed model (LFMM) [38] and Samβada [39]. Fourth, *F*_ST_ outliers were detected using genome scan methods including BAYESCAN [40] and DFDIST [41]. Fifth, *F*_ST_ outliers identified using both BAYESCAN and DFDIST were further examined for their correlations with environmental variables using a Bayesian logistic regression approach [42]. Sixth, we assessed the linear fit of environmental variables with the ordination axes derived from a redundancy analysis (RDA) on the outlier epigenetic variation [43]. Subsequently, the environmental variables that showed a significant linear fit to the RDA ordinations were used in a partial RDA (pRDA) to examine the correlation between the environmental variables and pRDA axes conditioned on geographic effect. Lastly, we tested whether epigenetic IBE is present when controlling for either the geographic or genetic effect. The main objectives of the present study were to (1) assess the inter-population relationship between genetic and epigenetic variation in natural populations of *Z. kawagoii*, (2) understand how environmental variables influence population evolutionary epigenetic divergence and local adaptation*,* and (3) investigate if there is an environmentally dependent epigenetic divergence when controlling for the presence of any genetic structure.

## 2. Materials and Methods

### 2.1. Sample Collection and Epigenotyping

**Figure 1:**
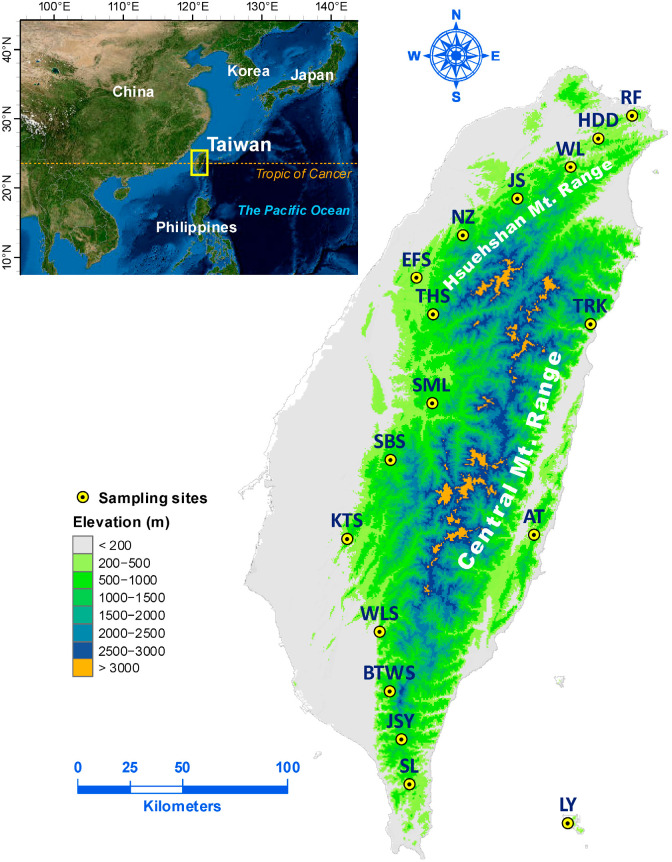
Sampling localities of the 17 *Zingiber kawagoii* populations. The map was derived from the default map database in ArcGIS v.10.8.1. Sampling site coordinates were used to depict population locations using ArcGIS. The elevation gradient was generated with a 20 m digital elevation model. See Table 1 for abbreviations of the population names.

This study used the same 17 populations of *Z. kawagoii* examined in an earlier investigation [23] (Figure 1). Genomic DNA was extracted from silica gel dried leaf samples using a cetyltrimethyl ammonium bromide (CTAB) procedure [44], and ethanol-precipitated DNA was dissolved in 200 µL TE buffer (pH 8.0). The 211 plants used in this study included all but one individual from the TRK population used in the earlier AFLP investigation [23] due to a technical problem. A NanoDrop spectrophotometer (NanoDrop Technology, Wilmington, DE, USA) was used to quantify total genomic DNA. In brief, total genomic DNA (200 ng) was digested with the methylation-sensitive enzymes *Hpa*II (1 U) and *Msp*I (1 U) as frequent cutters separately with rare cutter *EcoR*I (1 U). Restriction digestion was performed in a total 10 µL reaction volume with 10X CutSmart buffer (New England Biolabs, Ipswich, MA, USA) for 1.5 h at 37 °C and then deactivated at 65 °C for 15 min. Restricted DNA products were ligated to MSAP adaptors (5 µM) with 5 U T4 DNA ligase (Thermo Scientific, Vilnius, Lithuania) and 5X ligation buffer (Thermo Scientific) at 22 °C for 1 h in a 10 µL ligation reaction mixture.

Pre-selective amplification was performed using a polymerase chain reaction (PCR). Adaptor ligated product (1∶9 dilution with ddH_2_O) was mixed with 12 µM *Eco*RI primer (E00, Table S1), 12 µM *Hpa*II-*Msp*I primer (HM00, Table S1), 2.5 mM dNTPs, 1 U *Taq* DNA polymerase (Zymeset Biotech, Taipei, Taiwan), and 10X PCR buffer (Zymeset) in a 20 µL total volume. Pre-selective amplification was performed with an initial holding at 72 °C for 2 min and pre-denaturation at 94 °C for 3 min, followed by 25 cycles of 30 s at 94 °C, 30 s at 56 °C, and 1 min at 72 °C, with a final 5 min holding at 72 °C. Nine primer pair combinations with additional selective bases added at the ends of E00 and HM00 were used for selective amplification (Table S1). Fluorescent-dye-labeled 10 µM *Eco*RI selective primer was mixed with 10 µM *Hpa*II-*Msp*I primer, 2.5 mM dNTPs, 2 U *Taq* DNA polymerase (Zymeset), 10X PCR buffer (Zymeset), and diluted pre-selective amplified products (1∶19 dilution with ddH_2_O) in a 20 µL total volume. Subsequently, selective PCR was performed with an initial holding at 94 °C for 3 min, followed by 13 cycles of 30 s at 94 °C, 30 s at 65–56 °C (decreasing the temperature by 0.7 °C each cycle), 1 min at 72 °C, and then 23 cycles of 30 s at 94 °C, 30 s at 56 °C, and 1 min at 72 °C, with a final 5 min holding at 72 °C. Selective amplification products were electrophoresed with an ABI 3730XL DNA analyzer (Applied Biosystem, Foster City, CA, USA).

We scored amplification fragments in the range of 150–500 bp, setting a fluorescent threshold of 150 units using Peak Scanner v.1.0 (Applied Biosystem). Low peak fragments and those scored higher than 99% or less than 1% of individuals were removed. Additionally, fragments within one nucleotide in a ± 0.8 base pair window were recognized as the same fragment. To determine the DNA methylation status of every locus, the “mixed scoring 1” transformation scheme in the R function *MSAP-calc* [45] in the R environment [46] was applied to obtain mMSAP (methylated) and uMSAP (unmethylated) datasets. Three randomly chosen samples in each population for each primer combination were used to assess the epigenotyping error rate. The error rate for *Eco*RI-*Msp*I (e*Msp*I), *Eco*RI-*Hpa*II (e*Hpa*II), and a combined error rate (e*Msp*I + e*Hpa*II − 2e*_Msp_*_Ie*Hpa*II_) for each primer combination was calculated [27]. We removed loci with an error rate per locus greater than 5% [47]. The mean error rate was 2.15 and 2.12%, respectively, for e*Msp*I and e*Hpa*II, with a combined error rate of 4.18% (Table S1).

### 2.2. Environmental Variables

Sixteen environmental variables were included in this study [23]. These variables were annual temperate range (BIO7), mean temperature in the driest quarter (BIO9), annual precipitation (BIO12), precipitation in the coldest quarter (BIO19), aspect, elevation, slope, cloud cover (CLO), enhanced vegetation index (EVI), leaf area index (LAI), normalized difference vegetation index (NDVI), annual moisture index (MI), annual total potential evapotranspiration (PET), relative humidity (RH), soil pH, and mean wind speed (WS_mean_) (Table S2).

### 2.3. Epigenetic Diversity, Differentiation, and Clustering

The software AFLP-SURV v.1.0 [48] was used to estimate population unbiased expected heterozygosity (*uH*_E_) [49] and the percentage of polymorphic loci (*PPL*) based on allele frequencies using the settings of the Hardy–Weinberg equilibrium and a non-uniform prior distribution. Per locus *uH*_E_ was estimated using ARLEQUIN v.6.0 [50]. A linear mixed effect model (LMM) was used to estimate the difference in mean *uH*_E_ per locus between three types of markers (AFLP, mMSAP, and uMSAP). AFLP data were obtained from an earlier study [23]. In LMM, the marker type and population were used as a fixed factor and a random factor, respectively, and analyzed using the *lmer* function in the R package lme4 [51]. Significance was tested using the *Anova* function in the R package car based on type II Wald *χ*^2^ statistic [52]. Tukey’s post hoc pairwise comparisons of mean *uH*_E_ per locus between marker types were assessed using the *lsmeans* function in the R package emmeans [53]. Additionally, a paired *t*-test was used to assess the differences in mean *uH*_E_ between the three marker types at the population level. Population genetic (AFLP) and epigenetic (mMSAP and uMSAP) distance matrices were calculated using Nei’s genetic distance [54] using the *nei.dist* function in the R package poppr [55]. Mantel correlations for the inter-population relationship between genetic and epigenetic variation, based on these distance matrices, were assessed using the *mantel* function in the R package vegan [56]. A partial Mantel test, performed using the *mantel.partial* function in the R package vegan, was also used to assess the inter-population relationship between genetic and epigenetic variation controlling for the geographic effect. The geographic effect was computed using the coordinates of the sample sites.

Analysis of molecular variance (AMOVA) was used to estimate the level of genetic differentiation between populations (*Φ*_ST_) using the *poppr.amova* function in the R package poppr. Significance was tested using the *randtest* function in the R package ade4 [57] with 9999 permutations. Pairwise population *F*_ST_ was computed using ARLEQUIN, and significance was tested with 10,000 permutations. Pairwise population *F*_ST_ values were used to calculate the level of divergence for each population from the remaining populations as the mean value of pairwise *F*_ST_ for each population against the rest of the populations (denoted as the population mean *F*_ST_). Epigenetic homogeneous groups of individuals were assessed using discriminant analysis of principal components (DAPC) [58]. The *find.clusters* and *dapc* functions in the R package adegenet [59] were used in DAPC analysis setting *K* = 1–10. The Bayesian information criterion (BIC) in DAPC was estimated to determine the optimal number of clusters.

### 2.4. Test for F_ST_ Outliers

To detect signatures in selection on MSAP loci, *F*_ST_ outliers were identified using BAYESCAN and DFDIST. BAYESCAN v.2.1 [40] was used to estimate the ratio of posterior probabilities of selection over neutrality (the posterior odds (PO), log_10_(PO)). Two hundred pilot runs of 50,000 iterations followed by a sample size of 50,000 with a thinning interval of 20 among 10^6^ iterations were performed in BAYESCAN. A logarithmic scale of log_10_(PO) > 1 was used as strong evidence (posterior probability > 0.91) for selection over neutrality for a locus under directional selection [60]. DFDIST estimates a distribution of observed *F*_ST_ versus *uH*_E_, and loci under selection were identified by comparing them to a simulated neutral distribution. In DFDIST, we set critical frequency = 0.99, Zhivotovsky parameter = 0.25, trimmed mean *F*_ST_ = 0.3 (excluding 30% of highest and 30% of lowest *F*_ST_ values), smoothing proportion = 0.04, 500,000 resamplings, and critical *p* = 0.05. MSAP loci with observed *F*_ST_ against *uH*_E_ falling above the 95% confidence level of the simulated distribution were recognized as *F*_ST_ outliers under directional selection.

### 2.5. Test for Associations between Epigenetic Loci and Environmental Variables

The associations between all epigenetic loci and environmental variables were estimated using LFMM and Samβada. In LFMM, we considered population epigenetic structure as a random factor using a latent factor of 1 and 4, respectively, according to the DAPC results (see Results) for mMSAP and uMSAP. Matrices of mMSAP and uMSAP variation were used as fixed factors. Ten LFMM runs with 10,000 iterations of the Gibbs sampling algorithm and a burn-in period of 5000 cycles were performed for each environmental predictor. Z-scores for each environmental predictor were obtained by combining the results of ten independent LFMM runs, and *p*-values were adjusted using the genomic inflation factor [38]. Moreover, a 1% false discovery rate (FDR) was used to adjust the *p*-value using the *qvalue* function in the R package qvalue [61]. A multiple univariate logistic regression approach in Samβada was used to assess the correlations between the allele frequencies of mMSAP and uMSAP loci and the values of the environmental variables. Both Wald and G scores [39] were used, applying a 1% FDR for the *p*-value adjustment, to assess the fit of the model with environmental variables against the null model without environmental variables.

Moreover, a Bayesian logistic regression analysis, implemented with the *stan_glm* function in the R package rstanarm [42], was used to further justify the associations between environmental variables and the potential *F*_ST_ outliers that were identified with both BAYESCAN and DFDIST. The weakly informative priors following a student’s *t* distribution with mean zero and seven degrees of freedom were used in *stan_glm,* and the scale of the prior distribution was 10 for the intercept and 2.5 for the predictors. We ran all the *stan_glm* models using 4 chains, each containing 2000 warm-up and 2000 sampling steps, and a 95% credible interval was determined using the *posterior_interval* function in the R package rstanarm. In the *stan_glm* analysis, we obtained values for the effective sample size and convergence diagnostic statistic representing good priors applied and stable estimates obtained for each predictor.

### 2.6. Linear Relationships between Environmental Variables and the Ordination Axes of the Redundancy Analysis

A multivariate approach in an RDA analysis [43] was used to estimate the extent to which outlier variation was explained by the environmental variables. We explored the relationships between epigenetic variation and environmental drivers by fitting variables onto ordinations using the *envfit* function in the R vegan package. In the *envfit* analysis, a full RDA model was used to estimate the independent effect of the environment (16 environmental variables) fitting to the amount of outlier variation with 999 permutations and represented by the squared correlation coefficients (*R*^2^). All *p*-values were adjusted for multiple comparisons using 5% FDR. Environmental variables with significant fit to the outlier epigenetic variation were then used in a pRDA analysis. pRDA was analyzed in order to assess the correlations between environmental variables and the first two axes conditioned on the geographic effect. Significance of the pRDA ordination axes was assessed using the *anova.cca* function in the R package vegan with 999 permutations. The arrows pointed in the direction of maximum variation in the value of environmental variables, and the degree to which the variables correlated with pRDA axes was represented by the length of the arrows.

### 2.7. Epigenetic Isolation-by-Environment Controlling for Geographic or Genetic Effects

The Mantel function in the R package vegan (999 permutations) was used to test IBD based on the genetic and epigenetic distance matrices against the geographic distance matrix calculated using the *dist* function in R. To test IBE, Mantel and partial Mantel tests (controlling for the geographic effect) were used to assess the relationship between the epigenetic and environmental distance matrices, respectively, using the *mantel* and *mantel.partial* functions implemented in the R package vegan. IBD and IBE were also performed using the MMRR (multiple matrix regression with randomization) function in R [21]. In addition, IBE was also tested for epigenetic distance matrices against the environmental distance matrix controlling for genetic effect (AFLP) using the partial Mantel test and MMRR. This was performed to test if environmental variables influence epigenetic variation independent of the genetic effect (isolation-by-genetic structure, IBG). In MMRR, regression coefficients for IBD (*β*_D_), IBG (*β*_G_), and IBE (*β*_E_) were obtained, and the significance was determined after 999 permutations.

## 3. Results

### 3.1. Epigenetic Diversity and Structure

Overall, 9 primer pairs resolved a total of 481 unambiguous bands ranging from 22 to 107 (Table S1), and the number of loci estimated using the R *MSAP-calc* script [45] were 424 and 354, respectively, for mMSAP and uMSAP. The average *PPL* was 58.8% in mMSAP and 33.6% in uMSAP. The average *uH*_E_ was 0.160 in mMSAP and 0.119 in uMSAP (Table 1). The LMM analysis showed a significant difference in mean *uH*_E_ per locus among the three types of markers (AFLP, mMSAP, and uMSAP; *χ*^2^ = 2002.8, *p* < 0.0001). Pairwise comparisons between marker types revealed that the mean *uH*_E_ per locus was significantly smaller in AFLP (mean *uH*_E_ per locus = 0.104) compared to mMSAP (mean *uH*_E_ per locus = 0.223) and uMSAP (mean *uH*_E_ per locus = 0.131) (Table S3). At the population level, genetic *uH*_E_ was not significantly different from uMSAP epigenetic *uH*_E_ (paired *t*-test: *t*_16_ = 0.796, *p* = 0.438), but it was significantly lower compared to mMSAP epigenetic *uH*_E_ (paired *t*-test, *t*_16_ = −7.426, *p* < 0.0001). mMSAP *uH*_E_ was significantly higher compared to uMSAP *uH*_E_ (paired *t*-test, *t*_16_ = 8.007, *p* < 0.0001).

**Figure 2:**
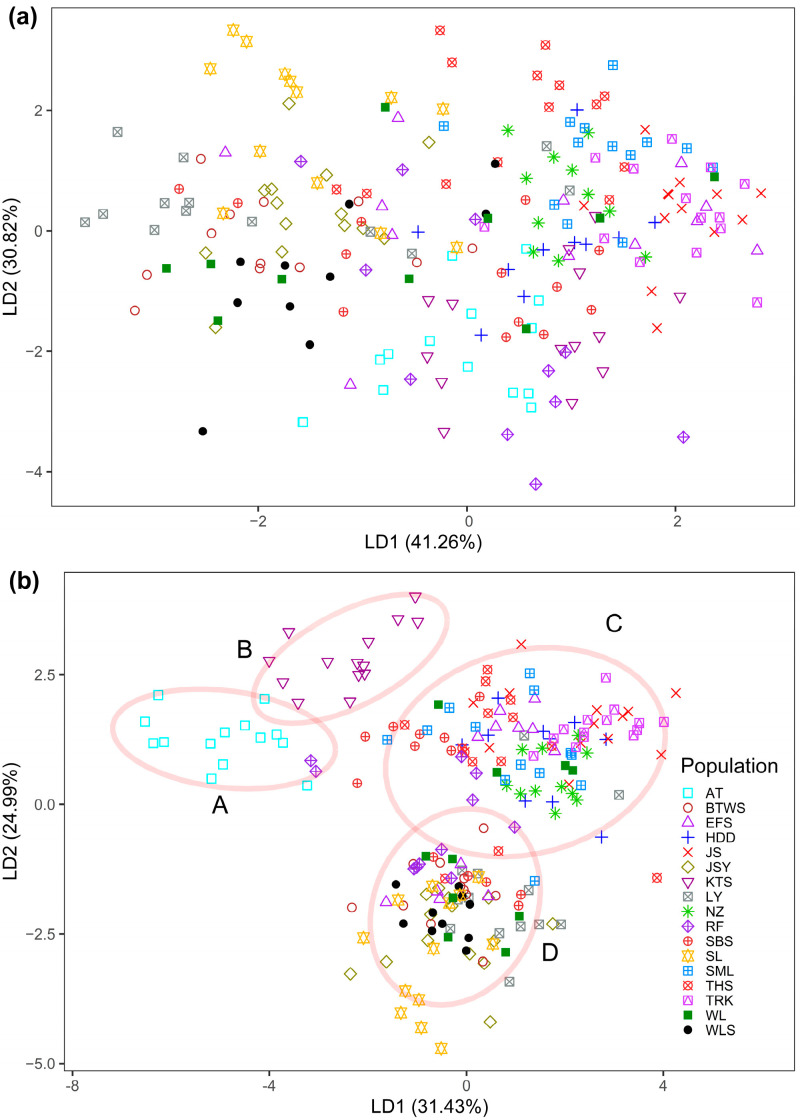
Analysis of epigenetic homogeneous groups of 211 individuals of *Zingiber kawagoii* based on the total (**a**) mMSAP and (**b**) uMSAP variation using DAPC. The first two linear discriminants described 72.08 and 56.42% of the total mMSAP and uMSAP variation, respectively.

The overall values of *F*_ST_ based on all loci were 0.044 and 0.106, respectively, for mMSAP and uMSAP (Table S4). The AMOVA showed an overall population differentiation (*Φ*_ST_) of 0.071 and 0.181, respectively, for mMSAP and uMSAP based on the total data (Table 2). The average *F*_ST_ values computed were 0.044 and 0.106, respectively, for the total mMSAP and uMSAP variation (Table S4). Using the total data, the lowest BIC was found at *K* = 2 and *K* = 6, respectively, for mMSAP and uMSAP in DAPC (Figure S1). No distinct mMSAP population clustering was found (Figure 2a), but four uMSAP population clusters were revealed (Figure 2b). However, individuals in the same population may be grouped in different uMSAP clusters. Unlike AFLP [23], the annual temperature range and latitude showed no significant relationship with the mMSAP population mean *F*_ST_ (Pearson’s *r* = −0.173, *p* = 0.507 and Pearson’s *r* = 0.031, *p* = 0.904, respectively) or with the uMSAP population mean *F*_ST_ (Pearson’s *r* = −0.046, *p* = 0.860 and Pearson’s *r* = −0.020, *p* = 0.938, respectively).

### 3.2. Inter-Population Relationship between Genetic and Epigenetic Variation

**Figure 3:**
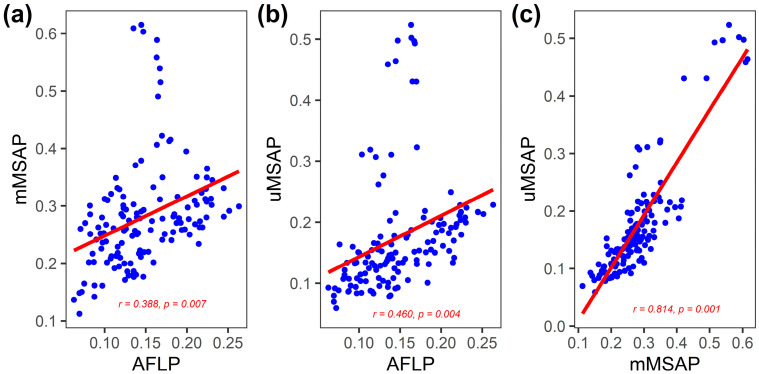
Inter-population relationships between genetic (AFLP) and epigenetic variation (mMSAP and uMSAP) in the 17 *Zingiber kawagoii* populations. Linear relationships between distance matrices of (**a**) AFLP vs. mMSAP, (**b**) AFLP vs. uMSAP, and (**c**) mMSAP vs. uMSAP are depicted. The Mantel *r* and *p*-values for the partial Mantel test are annotated.

A pairwise population Nei’s distance matrix was used in a Mantel test to investigate the inter-population correlation between genetic and epigenetic variation. We found significant inter-population correlations between genetic and epigenetic distance (AFLP vs. mMSAP: Mantel *r* = 0.484, *p* = 0.002; AFLP vs. uMSAP: Mantel *r* = 0.596, *p* = 0.001; and mMSAP vs. uMSAP: Mantel *r* = 0.832, *p* = 0.001), and the linear fitting lines are displayed in Figure 3. The relationships between the three marker types were also significant when the effect of geography was excluded using the partial Mantel test (AFLP vs. mMSAP: Mantel *r* = 0.388, *p* = 0.007; AFLP vs. uMSAP: Mantel *r* = 0.460, *p* = 0.004; and mMSAP vs. uMSAP: Mantel *r* = 0.814, *p* = 0.001).

### 3.3. F_ST_ Outlier Identification and Association between Outliers and Environmental Variables

In the DFDIST analysis, 41 mMSAP and 10 uMSAP loci (9.67 and 2.82%, respectively) were identified as being *F*_ST_ outliers (Figure S2). The BAYESCAN analysis identified 45 mMSAP and 15 uMSAP loci as being *F*_ST_ outliers, corresponding to 10.61 and 4.24% of all loci, respectively. Although we set log_10_(PO) > 1.0 as a criterion for strong evidence in the identification of outliers using BAYESCAN, most of the outliers identified with BAYESCAN had a log_10_(PO) > 2 (decisive evidence for selection corresponding to a probability > 0.99) [58], except two mMSAP outliers (mX06HM_2011 and mX10HM_4950) (Table S5). A total of 10 mMSAP and 9 uMSAP loci, respectively, were identified as outliers using both DFDIST and BAYESCAN (Table S5, Figure S2). No outlier mMSAP loci were correlated with BIO19 or CLO assessed using LFMM, Samβada, and rstanarm (Table S5). Most outlier loci exhibited a significant association with two or more environmental variables based on the three regression approaches (Table S5). The *Φ*_ST_ was 0.284 in mMSAP and 0.404 in uMSAP based on the outlier loci (Table 2).

### 3.4. Environmental Effect on Outlier Epigenetic Variation

**Figure 4:**
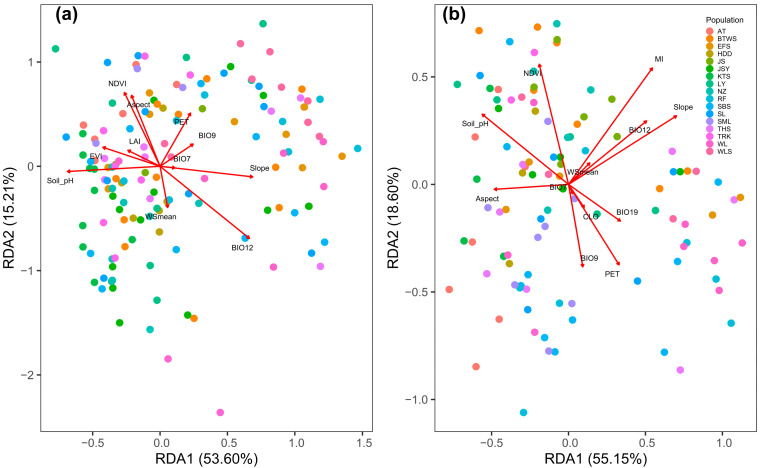
Partial RDA analysis using (**a**) mMSAP and (**b**) uMSAP variation explained by the effects of the environment conditioned on the geographic effect. The colored points refer to individuals (colors represent which population they were sampled from), and the red vectors represent environmental predictors. The length and direction of vectors indicate relative correlation strength of the environmental predictors with RDA axes.

Using the 16 environmental variables in the full RDA model, the statistical test supported the role of the environment in shaping the distribution of the outlier mMSAP and uMSAP epigenotypes (mMSAP: adjusted *R*^2^ = 0.273, *F* = 5.917, *p* = 0.001; uMSAP: adjusted *R*^2^ = 0.390, *F* = 9.414, *p* = 0.001). The full RDA model analysis with envfit suggested that outlier mMSAP and uMSAP variation, respectively, correlated significantly with 11 and 12 environmental variables (5% FDR adjusted *p* < 0.05, Table 3). These 11 and 12 environmental variables were used, respectively, in the pRDA model for outlier mMSAP and uMSAP conditioned on geography. The first two axes of the pRDA explained 68.81 and 73.75% of the outlier mMSAP and uMSAP variation, respectively (mMSAP: adjusted *R*^2^ = 0.140, *F* = 4.427, *p* = 0.001; uMSAP: adjusted *R*^2^ = 0.269, *F* = 8.224, *p* = 0.001) (Figure 4).

When conditioned on the geographic effect, the outlier mMSAP variation was highly correlated with slope, soil pH, and BIO12 on the first pRDA axis (Table 4 and Figure 4). Aspect, NDVI, PET, and BIO12 were highly correlated with the outlier mMSAP variation on the second pRDA axis. On the first pRDA axis, the outlier uMSAP variation showed strong correlations with aspect, slope MI, soil pH, and BIO12. The second pRDA axis revealed high correlations between MI and NDVI and the outlier uMSAP variation. The annual temperature range had a relatively higher *R*^2^ (mMSAP: *R*^2^ = 0.308; uMSAP: *R*^2^ = 0.129) than the other environmental variables in the envfit analysis (Table 3). However, the annual temperature range had low levels of correlation with the outlier mMSAP (RDA1 biplot score = 0.074, RDA2 biplot score = −0.010) and uMSAP (RDA1 biplot score = 0.019, RDA2 biplot score = −0.029) variation in both pRDA axes when controlling for the geographic effect (Table 4 and Figure 4).

### 3.5. Contribution of Environment to Explaining the Total and Outlier Epigenetic Variation

The Mantel test revealed a significant genetic IBD based on the total AFLP data (Mantel *r* = 0.457, *p* = 0.001) [23]. A significant epigenetic IBD was also found based on the total mMSAP and uMSAP using the Mantel test (mMSAP: Mantel *r* = 0.126, *p* = 0.001; uMSAP: Mantel *r* = 0.184, *p* = 0.001) and MMRR (mMSAP: *r* = 0.112, *p* = 0.001; uMSAP: *r* = 0.174, *p* = 0.001) (Table 5). A significant IBE was found in all analyses based on the total variation using the Mantel test (mMSAP: Mantel *r* = 0.148, *p* = 0.002; uMSAP: Mantel *r* = 0.195, *p* = 0.001) and MMRR (mMSAP: *r* = 0.131, *p* = 0.002; uMSAP: *r* = 0.184, *p* = 0.001). Moreover, a significant IBE can be inferred based on the total (mMSAP: Mantel *r* = 0.121, *p* = 0.013; uMSAP: Mantel *r* = 0.150, *p* = 0.001) and outlier (mMSAP: Mantel *r* = 0.191, *p* = 0.001; uMSAP: Mantel *r* = 0.184, *p* = 0.001) datasets when controlling for geography using the partial Mantel test. MMRR also revealed a significant IBE when controlling for the geographic effect based on the total (mMSAP: *R*^2^ = 0.030, *β*_E_ = 0.110, *p* = 0.012 vs. *β*_D_ = 0.084, *p* = 0.002; uMSAP: *R*^2^ = 0.056, *β*_E_ = 0.145, *p* = 0.001 vs. *β*_D_ = 0.130, *p* = 0.001) and outlier (mMSAP: *R*^2^ = 0.098, *β*_E_ = 0.128, *p* = 0.001 vs. *β*_D_ = 0.138, *p* = 0.001; uMSAP: *R*^2^ = 0.068, *β*_E_ = 0.132, *p* = 0.001 vs. *β*_D_ = 0.100, *p* = 0.001) variation.

Partial Mantel tests revealed strong correlations between mMSAP and uMSAP variation and environmental heterogeneity when controlling for genetic structure based on the total (mMSAP: Mantel *r* = 0.091, *p* = 0.031; uMSAP: Mantel *r* = 0.121, *p* = 0.001) and outlier (mMSAP: Mantel *r* = 0.150, *p* = 0.001; uMSAP: Mantel *r* = 0.191, *p* = 0.001) data. However, MMRR revealed non-significant IBE based on the total mMSAP variation when controlling for genetic structure (*R*^2^ = 0.036, *β*_E_ = 0.086, *p* = 0.051 vs. *β*_G_ = 0.138, *p* = 0.009), but a significant IBE was found when controlling for genetic structure based on the total uMSAP variation (*R*^2^ = 0.062, *β*_E_ = 0.121, *p* = 0.001 vs. *β*_G_ = 0.190, *p* = 0.001). Nonetheless, MMRR revealed a significant IBE based on the outlier mMSAP and uMSAP variation when controlling for the genetic structure (mMSAP: *R*^2^ = 0.102, *β*_E_ = 0.105, *p* = 0.002 vs. *β*_G_ = 0.182, *p* = 0.001; uMSAP: *R*^2^ = 0.070, *β*_E_ = 0.136, *p* = 0.001 vs. *β*_G_ = 0.122, *p* = 0.001).

## 4. Discussion

Theoretical work has shown that beneficial and heritable epigenetic variants may play important roles for populations to adapt quickly to local conditions independent of genetic changes [7]. Although empirical studies have shown epigenetics may act independently from underlying genetic variation [29,30,33,62,63], genotypes and epigenotypes may interact and together affect biological processes, for example, in *Viola cazorlensis* [27], *Arabidopsis thaliana* [28], and *Taiwania cryptomerioides* [64]. Our results indicate inter-population correlations between genetic and epigenetic variation, even with the exclusion of the geographic effect using the partial Mantel test (Figure 3). The underlying mechanism could be that the epigenetic variation interplay with genetic variation and epigenetic variation is at least in part a downstream, subsidiary effect of genetic changes [6,16,17,18]. Epigenetic variation may have played a role in speeding up the local adaptation of *Z. kawagoii*. Relatively lower *Z. kawagoii* AFLP diversity was found compared to that of *Zingiber* species distributed in Brazil and India [23]. We found that mean mMSAP diversity is relatively higher compared to mean AFLP diversity (Table 1, cf. 23). At the population level, most populations of *Z. kawagoii* had a relatively higher level of epigenetic than genetic diversity, particularly when compared between AFLP and mMSAP (Table 1, cf. 23). Additionally, mean *uH*_E_ per locus was significantly smaller in AFLP compared to mMSAP and uMSAP (Table S3). These results suggest that epigenetic mutation may occur at a faster rate than genetic mutation [65,66]. Moreover, lower epigenetic than genetic differentiation (Table 2 and Table S4; cf. 23) suggests larger epigenetic variation in shorter spatial distances might contribute considerably to population epigenetic diversity [27,33,34,67,68]. It is possible that epigenetic variation may provide a compensating source for the low level of genetic variation [7,9,10].

Neutral epigenetic divergence may occur via drift, and inter-population correlation between genetic and epigenetic variation may involve no functional link [5,69]. The higher genetic than epigenetic differentiation found in *Z. kawagoii* and other plants [30,63,64,70] (Table 2 and Table S4; cf. 23) suggests that geographic distance was a better predictor of genetic than epigenetic differentiation. This is evidenced by the larger Mantel statistic for AFLP relative to mMSAP and uMSAP in this study (AFLP: Mantel *r* = 0.457; mMSAP: Mantel *r* = 0.126; uMSAP: Mantel *r* = 0.184). Nonetheless, significant epigenetic IBD was detected with both the Mantel test and MMRR based on the total and outlier data (Table 5), suggesting that population epigenetic variation may be partly caused by random epigenetic drift [71]. Additionally, the stronger signal in genetic IBD than in epigenetic IBD suggests that the signal is biased by non-unidirectional changes in environmental gradients, which may impede epigenetic IBD [72,73].

Epigenetic changes can be triggered by biotic and abiotic stresses in natural environments and provide potential for novel heritable variation [74]. Environmental changes affect epigenetic variation in many organisms, which might help them adapt to rapid environmental changes [27,28,29,30,33]. Thus, the role of epigenetics in response to environmental stimulus has been an important issue in evolution [36]. With a significant inter-population correlation between genetic and epigenetic variation, strong epigenetic IBE can still be found when controlling for the effect of any genetic structure using both the partial Mantel test and MMRR (Table 5), particularly based on the outlier data. It is likely that part of the epigenetic variation is not coupled with genetic changes, and environments may act as causal factors in inducing epigenetic variation. Thus, epigenetic variation may play a role in the rapid evolution of individuals to various environments [64]. Epigenetic profiles may diverge between environments contributing to differentially induced effects [29,75]. Additionally, there was a significantly lower level of mean genetic *uH*_E_ per locus compared to mean epigenetic *uH*_E_ per locus (Table S3), reinforcing the role of epigenetic variation in *Z. kawagoii* adaptation. This may enable higher survival in a population encountering novel environments until the acquisition of beneficial genetic mutation.

The current study extends the earlier research [23] by examining the links between environmental variables and epigenetic variants. Unlike the earlier study [23], which showed that annual temperature range was the main driver for latitudinal adaptive divergence, this study found that the annual temperature range may play only a minor role in the adaptive epigenetic divergence, as suggested by low biplot scores in the first and second pRDA axes (Table 4 and Figure 4). This suggests low correlation between the annual temperature range and the outlier MSAP variation at larger spatial scales. Moreover, epigenetics in this study, similar to the earlier genetic study [23], indicated that combinations of different environmental drivers may act as selective pressures in invoking adaptive evolution. However, environmental variables may have differential effects on adaptive genetic and epigenetic variation in *Z. kawagoii*. The results of pRDA analyses in this study suggest outlier mMSAP variation is associated mainly with slope, soil pH, NDVI, PET, and BIO12, whereas outlier uMSAP variation is mainly associated with aspect, slope, MI, NDVI, soil pH, and BIO12 (Table 4 and Figure 4). However, the contributions of various environmental variables with high correlations with both pRDA axes (Table 4 and Figure 4) suggest that these variables may have acted in concert on various genes invoking adaptive epigenetic divergence.

Although it is unclear whether environmentally dependent epigenetic changes at distinct loci are the direct consequence of environments, local environments may play a role in mediating heritable epigenetic divergence for certain loci that are responsive to environmental conditions [36,72,76]. This may be advantageous if epigenetic changes that are stress responsive and are ecologically relevant. For example, individuals of *L. racemosa* from salt marsh populations display a shrub-like phenotype with lower levels of DNA methylation, whereas individuals of riverside populations with a tree-like phenotype had higher levels of DNA methylation [34]. In this mangrove species, genes may be activated via demethylation due to salinity stress in the salt marsh populations in contrast to hypermethylation of these genes in the riverside populations. Correlations between epigenetic changes and traits related to plant growth and development can be found, for example, leaf traits in *Prunus mume* [77] and *Carpobrotus edulis* [78], flower morphology in *V. cazorlensis* [27], and inhabiting different natural habitats in *Phragmites australis* and *Lilium bosniacum* [79,80]. Epigenetic changes are closely related to climatic factors such as temperature [31,33,81] and precipitation [31,33]. Epigenetic changes are also found to be closely associated with ecological factors such as soil factors [31,82], NDVI, and PET [31,33]. Topographic variables including slope and aspect are also found to be closely related to epigenetic changes [31,33]. Therefore, environmentally dependent epigenetic changes may be related to the growth and development of plants, thus contributing to ecological and evolutionary processes in natural populations.

## 5. Conclusions

Genetic adaptation to environmental changes is crucial for the conservation and survival of species. In addition, researchers in evolutionary biology are increasingly interested in epigenetic adaptations to different environments. Both genetic and epigenetic variations may be sources of variation that play important roles in adapting to environmental heterogeneity and, hence, are important for understanding how the environment shapes natural population diversity in a changing environment. In the present study, epigenetic variation provided no distinct regional substructuring, particularly based on the total mMSAP data. This study revealed low epigenetic population differentiation, indicating that larger epigenetic variation occurs mainly at shorter spatial scales. Epigenetic variation may have compensated for the lack of genetic variation and played an important role in the adaptive divergence and local adaptation in *Z. kawagoii* populations. We identified that outlier MSAP loci associated strongly with specific environmental variables that may have played important roles in the local adaptation and survival of *Z. kawagoii* populations despite significant IBD. The local adaptation involving epigenetic changes in *Z. kawagoii* is also evidenced by the finding of significant IBE based on the outlier epigenetic variation when controlling for the geographic or genetic effects. Nonetheless, this study suggests that selection may be involved in population divergence at epigenetic sites that are partly linked to genetic sites in *Z. kawagoii*. Our findings highlight that methylation changes can be substrates for natural selection associated with environmental variables and may have a long-term consequence on the adaptation and survival of *Z. kawagoii* populations. This study also emphasizes the importance of investigating population epigenetic variation in association with environmental gradients apart from adaptive genetic divergence.

## Figures and Tables

**Table 1:** Site properties and epigenetic parameters of the 17 sampled populations of *Zingiber kawagoii* estimated using total mMSAP and uMSAP variation.

Population	Latitude/Longitude	Altitude (m)	*N*	mMSAP		uMSAP	
				*PPL* (%)	*uH*_E_ (SE)	*PPL* (%)	*uH*_E_ (SE)
Antong (AT)	23.2847/121.3721	610	14	45.5	0.159 (0.007)	31.9	0.112 (0.008)
Beitawushan (BTWS)	22.6148/120.7022	1192	12	83.0	0.190 (0.005)	34.7	0.140 (0.008)
Erfenshan (EFS)	24.3919/120.8240	769	12	77.8	0.179 (0.006)	30.5	0.133 (0.009)
Huangdidian (HDD)	24.9894/121.6799	432	10	59.7	0.158 (0.007)	38.1	0.109 (0.008)
Jianshi (JS)	24.7307/121.2895	850	13	32.1	0.120 (0.007)	26.3	0.093 (0.008)
Jinshuiying (JSY)	22.4075/120.7564	1488	14	54.2	0.167 (0.006)	37.3	0.150 (0.010)
Kantoushan (KTS)	23.2671/120.5010	583	14	42.2	0.146 (0.008)	31.1	0.096 (0.008)
Lanyu (LY)	22.0496/121.5257	302	13	46.0	0.152 (0.006)	33.1	0.136 (0.010)
Nanzhuang (NZ)	24.5742/121.0436	467	11	49.1	0.130 (0.008)	28.2	0.099 (0.008)
Ruifang (RF)	25.0861/121.8385	349	11	76.9	0.185 (0.006)	44.9	0.131 (0.008)
Shibishan (SBS)	23.6077/120.7045	1347	13	82.3	0.179 (0.006)	30.5	0.132 (0.010)
Shuangliu (SL)	22.2140/120.7961	255	13	75.0	0.174 (0.006)	30.2	0.128 (0.009)
Sunmoonlake (SML)	23.8519/120.8982	816	13	33.0	0.119 (0.007)	28.2	0.103 (0.008)
Tahsueshan (THS)	24.2326/120.9003	937	14	47.4	0.158 (0.007)	31.1	0.133 (0.010)
Taroko (TRK)	24.1880/121.6382	929	14	40.1	0.136 (0.007)	27.4	0.085 (0.007)
Wulai (WL)	24.8663/121.5498	143	10	72.9	0.166 (0.005)	45.2	0.134 (0.008)
Weiliaoshan (WLS)	22.8695/120.6571	694	10	81.6	0.206 (0.005)	42.4	0.099 (0.006)
Average			12.4	58.8	0.160	33.6	0.119

*N*, sample size; *PPL* (%), percentage of polymorphic loci; *uH*_E_, unbiased expected heterozygosity.

**Table 2:** Epigenetic differentiation between the 17 populations of *Zingiber kawagoii* based on the total and outlier mMSAP and uMSAP variation using an analysis of molecular variance (AMOVA).

Source of Variation	df	Sum of Squares	Percent Variation	*Φ* Statistic (*p*)
mMSAP				
Total data				
Between populations	16	1456.462	7.08	*Φ*_ST_ = 0.071 (0.0001)
Within populations	194	9083.036	92.92	
Total	210	10,539.498	100	
Outlier data				
Between species	16	131.396	28.39	*Φ*_ST_ = 0.284 (0.0001)
Within populations	194	269.249	71.61	
Total	210	400.645	100	
uMSAP				
Total data				
Between populations	16	1372.462	18.13	*Φ*_ST_ = 0.181 (0.0001)
Within populations	194	4442.694	81.87	
Total	210	5815.156	100	
Outlier data				
Between species	16	172.242	40.42	*Φ*_ST_ = 0.404 (0.0001)
Within populations	194	221.853	59.58	
Total	210	394.095	100	

*p*, *p*-value.

**Table 3:** Environmental covariates that were significantly associated with outlier epigenetic variation based on the *envfit* analysis. *R*^2^, the amount of variation explained by each covariate in the model, was determined using *envfit*. Adjusted *p*, the *p*-value corrected for multiple comparisons, was determined using 5% FDR after 999 permutation tests.

Environmental Variable	mMSAP		uMSAP	
	*R* ^2^	Adjusted *p*	*R* ^2^	Adjusted *p*
Aspect	0.127	0.002	0.106	0.002
Elevation	0.005	0.655	0.001	0.968
Slope	0.065	0.004	0.104	0.002
CLO	0.009	0.455	0.083	0.002
EVI	0.079	0.002	0.016	0.247
LAI	0.055	0.005	0.013	0.303
MI	0.004	0.739	0.230	0.002
NDVI	0.091	0.002	0.076	0.002
PET	0.039	0.016	0.189	0.002
RH	0.021	0.159	0.009	0.409
Soil pH	0.100	0.002	0.042	0.015
WS_mean_	0.201	0.002	0.090	0.002
BIO7	0.308	0.002	0.129	0.002
BIO9	0.101	0.002	0.097	0.002
BIO12	0.243	0.002	0.097	0.002
BIO19	0.002	0.805	0.057	0.002

**Table 4:** The pRDA biplot scores for constraining environmental variables associated with epigenetic variation controlling for the geographic effect.

Environmental Variable	mMSAP		uMSAP	
	RDA1	RDA2	RDA1	RDA2
Aspect	−0.141	0.454	−0.322	−0.015
Slope	0.459	−0.067	0.468	0.213
CLO			0.068	−0.072
EVI	−0.283	0.122		
LAI	−0.160	0.102		
MI			0.364	0.361
NDVI	−0.177	0.472	−0.127	0.373
PET	0.151	0.341	0.219	−0.249
Soil pH	−0.459	−0.033	−0.374	0.218
WS_mean_	0.038	−0.264	0.092	0.067
BIO7	0.074	−0.010	0.019	−0.029
BIO9	0.164	0.142	0.062	−0.256
BIO12	0.442	−0.461	0.338	0.197
BIO19			0.225	−0.114

**Table 5:** Isolation-by-environment (IBE), isolation-by-distance (IBD), and isolation-by-genetic structure (IBG) tested using the Mantel test, partial Mantel tests, and multiple matrix regression with randomization (MMRR). Euclidean distance matrices were generated based on the total and outlier mMSAP and uMSAP, AFLP, geography, and environment. The partial Mantel test and MMRR were used to infer the effects of IBE controlling for geography or genetic structure. In MMRR, *R*^2^ represents the total amount of variation explained by both geographic and environmental factors or by both genetic structure and environmental factors. Regression coefficients for IBD (*β*_D_), IBG (*β*_G_), and IBE (*β*_E_) were obtained using MMRR.

	Mantel Test		Partial Mantel Test					
	IBE	IBD	IBE controlling for geographic effect			IBE controlling for genetic effect		
	*r* (*p*)	*r* (*p*)	*r* (*p*)			*r* (*p*)		
Total data								
mMSAP	0.148 (0.002)	0.126 (0.001)	0.121 (0.013)			0.091 (0.031)		
uMSAP	0.195 (0.001)	0.184 (0.001)	0.150 (0.001)			0.121 (0.001)		
Outlier data								
mMSAP	0.243 (0.001)	0.253 (0.001)	0.191 (0.001)			0.150 (0.001)		
uMSAP	0.222 (0.001)	0.188 (0.001)	0.184 (0.001)			0.191 (0.001)		
**MMRR**								
	IBE	IBD	IBE controlling for geographic effect			IBE controlling for genetic effect		
	*r* (*p*)	*r* (*p*)	*R* ^2^	*β*_D_ (*p*)	*β*_E_ (*p*)	*R* ^2^	*β*_G_ (*p*)	*β*_E_ (*p*)
Total data								
mMSAP	0.131 (0.002)	0.112 (0.001)	0.030	0.084 (0.002)	0.110 (0.012)	0.036	0.138 (0.009)	0.086 (0.051)
uMSAP	0.184 (0.001)	0.174 (0.001)	0.056	0.130 (0.001)	0.145 (0.001)	0.062	0.190 (0.001)	0.121 (0.001)
Outlier data								
mMSAP	0.163 (0.001)	0.171 (0.001)	0.098	0.138 (0.001)	0.128 (0.001)	0.102	0.182 (0.001)	0.105 (0.002)
uMSAP	0.157 (0.001)	0.132 (0.001)	0.068	0.100 (0.001)	0.132 (0.001)	0.070	0.122 (0.001)	0.136 (0.001)

*r*, Mantel *r* statistic; *p*, *p*-value.

## Data Availability

The data presented in this study are available on request from the corresponding author.

## Supplementary Materials

The following are available online at https://www.mdpi.com/article/10.3390/plants12071558/s1, Figure S1: Bayesian information criterion for the evaluation of clustering scenarios analyzed based on mMSAP and uMSAP. Figure. S2: Venn diagrams showing the number of outlier MSAP (mMSAP and uMSAP) loci identified using genome scans of natural populations of *Zingiber kawagoii* using BAYESCAN and DFDIST. Table S1: Primer combinations, number of markers, and error rate per locus calculated using the MSAP technique. Table S2: The 16 environmental variables of the 17 populations of *Zingiber kawagoii*. Table S3: Summary of Tukey’s post hoc pairwise comparisons of the mean unbiased expected heterozygosity (*uH*_E_) per locus in *Zingiber kawagoii* between marker types (AFLP, mMSAP, and uMSAP) using a linear mixed effect model. Table S4: Matrix of pairwise *F*_ST_ (lower diagonal) and *p*-values (upper diagonal) based on the total mMSAP and uMSAP data of the 17 populations of *Zingiber kawagoii* estimated using ARLEQUIN. Table S5: *F*_ST_ outliers identified using BAYESCAN and DFDIST that were strongly associated with environmental variables.
