# Supplementary material for: The Contribution of Epigenetics to Evolutionary Adaptation in Zingiber kawagoii Hayata (Zingiberaceae) Endemic to Taiwan
Source: Plants (Basel). 2023 Apr 4;12(7):1558. doi: 10.3390/plants12071558 (PMC10096833; doi:10.3390/plants12071558)
Supplement: Supplementary file 1 [file plants-12-01558-s001.zip › plants-2277713-supplementary.pdf]

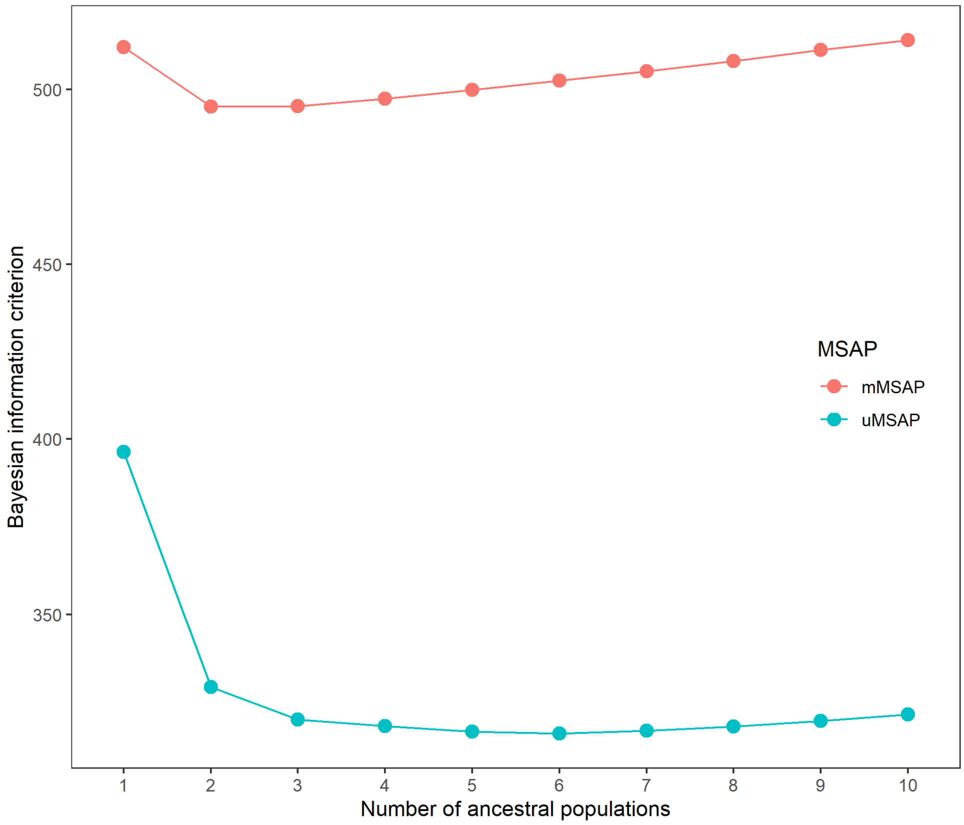

Figure S1. Bayesian information criterion for evaluation of clustering scenarios analyzed based on mMSAP and uMSAP.

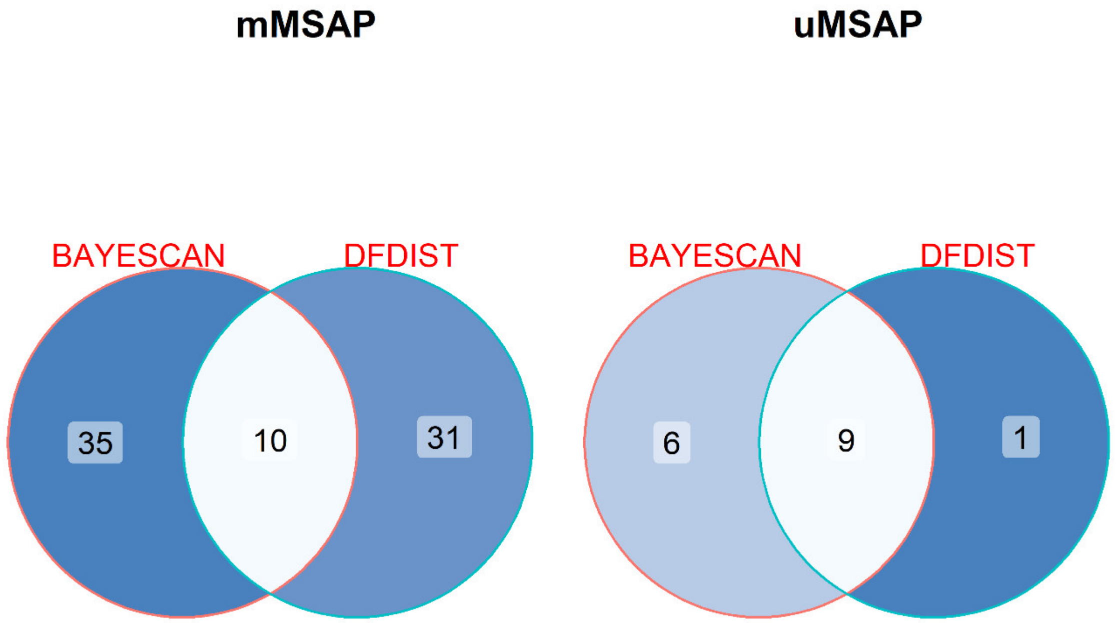

Figure. S2. The Venn diagrams showing the number of outlier MSAP (mMSAP and uMSAP) loci identified by genome scans of natural populations of *Zingiber kawagoii* using BAYESCAN and DFDIST. Numbers are presented for each separate group, and the intersection represents the number of loci identified by both genome scan methods.

Table S1. Primer combinations, number of markers, and error rate per locus calculated in MSAP technique.

**Primer Combination**	**Number of Markers**	**Number of mMSAP Markers**	**Number of uMSAP Markers**	**Error Rate (%)**		**Combined Error Rate (%)**
				***Hpa*II**	***Msp*I**	
1 E00 ACAGT + HM00 AGG	22	21	15	2.82	1.96	4.666
2 E00 ACAGT + HM00 CAG	38	35	26	2.89	2.05	4.823
3 E00 ACAGT + HM00 GCA	50	42	33	1.97	2.23	4.112
4 E00 ACAAG + HM00 CAG	107	90	81	1.47	1.85	3.262
5 E00 ACAAG + HM00 CGT	95	85	70	1.47	1.57	2.995
6 E00 ACAAG + HM00 CCC	30	26	20	1.99	2.20	4.109
7 E00 ACAAG + HM00 CTG	38	33	28	2.20	2.42	4.518
8 E00 ACAAG + HM00 TAC	68	63	56	2.15	2.42	4.466
9 E00 ACATA + HM00 TAC	33	29	25	2.40	2.41	4.694
	481 (53.4)	424 (47.1)	354 (39.3)	(2.15)	(2.12)	(4.18)

E00 (5′-GACTGCGTACCAATTC-3′)

HM00 (*Hpa*II-*Msp*I, 5′-ATCATGAGTCCTGCTCGG-3′)

Table S2. The 16 environmental variables of the 17 populations of *Zingiber kawagoii*. See **Table 1** for population code.

**Population**	**Aspectt**	**Elevation**	**Slope**	**CLO**	**EVI**	**LAI**	**MI**	**NDVI**	**PET**	**RH**	**Soil_pH**	**WS_{mean}**	**BIO7**	**BIO9**	**BIO12**	**BIO19**
AT	288.0	610.0	22.7	6.8	0.5	5.3	53.9	0.8	1380.9	78.2	6.0	3.1	15.6	16.4	1933.0	181.0
BTWS	120.1	1192.0	34.5	6.0	0.5	5.3	132.3	0.8	1497.8	76.7	4.9	2.7	14.9	15.5	4616.0	103.0
EFS	314.4	769.0	35.9	5.8	0.5	6.2	72.6	0.9	1634.2	78.2	4.2	2.7	18.1	17.6	2494.0	196.0
HDD	299.2	432.0	31.1	7.6	0.5	6.0	159.8	0.8	1419.2	78.9	4.6	2.6	18.4	16.3	3481.0	650.0
JS	165.0	850.0	35.3	6.7	0.5	6.1	185.1	0.9	1436.6	78.9	5.5	2.5	17.3	9.8	2539.0	337.0
JSY	81.0	1488.0	33.1	6.1	0.5	5.1	133.9	0.8	1450.0	75.9	4.9	2.7	14.3	13.2	4749.0	244.0
KTS	290.7	583.0	18.0	5.6	0.5	5.7	111.6	0.8	1622.9	78.7	6.3	2.7	17.0	17.8	3120.0	101.0
LY	235.6	302.0	22.8	7.6	0.5	5.7	156.4	0.8	1379.9	87.6	7.3	7.5	13.8	21.3	2760.0	426.0
NZ	269.4	457.0	21.9	6.1	0.4	6.1	124.6	0.8	1489.5	78.7	4.5	2.6	18.1	17.0	2564.0	278.0
RF	250.4	394.0	25.5	7.6	0.4	4.8	177.1	0.8	1398.8	78.3	4.6	2.8	18.4	16.7	3282.0	781.0
SBS	81.7	1347.0	28.9	6.0	0.5	6.3	142.6	0.8	1855.1	81.4	4.7	2.2	16.0	14.2	2726.0	141.0
SL	63.7	255.0	10.8	6.1	0.5	6.3	66.8	0.9	1830.7	76.2	5.4	3.0	14.4	19.2	3100.0	169.0
SML	256.7	816.0	21.4	6.2	0.5	5.7	82.2	0.8	1757.0	81.1	4.5	1.3	16.5	16.1	2262.0	162.0
THS	323.1	937.0	3.4	5.8	0.5	6.1	123.5	0.8	1632.2	78.3	4.3	2.5	17.1	14.5	2569.0	192.0
TRK	294.9	929.0	20.5	7.0	0.5	5.8	143.7	0.8	1453.3	78.8	6.5	2.8	16.5	13.6	2292.0	273.0
WL	105.7	143.0	29.3	7.3	0.4	5.5	129.7	0.8	1477.3	78.8	5.0	2.4	18.5	14.3	3231.0	481.0
WLS	358.4	694.0	36.7	5.8	0.5	6.0	141.7	0.8	1769.9	77.3	4.9	2.8	16.1	18.3	3093.0	89.0

Aspect (0–360°); Elevation (m); Slope (0–90°); BIO7, annual temperature range (°C); BIO9, Mean Temperature of Driest Quarter (°C); BIO12, annual precipitation (mm); BIO19, precipitation of the coldest quarter (mm); CLO, cloud cover (); EVI, enhanced vegetation index; LAI, leaf area index (m2/m2); MI, annual moisture index (unitless); NDVI, normalized difference vegetation index (unitless); PET, annual total potential evapotranspiration (kg/m2/year); RH, relative humidity (%); WS_{mean}, mean wind speed (m/s).

Table S3. Summary of Tukey's post-hoc pairwise comparisons of the mean unbiased expected heterozygosity (uH_E) per locus in *Zingiber kawagoii* between marker types (AFLP, mMSAP, and uMSAP) using a linear mixed effect model. In linear mixed effect model, marker type was treated as a fixed factor and population as a random factor.

	**Mean**	**SE**	**df**	***t***	***p***
AFLP vs. mMSAP	-0.1193	0.00270	24444	-44.128	< 0.001
AFLP vs. uMSAP	-0.0268	0.00286	24444	9.363	< 0.001
mMSAP vs. uMSAP	0.0925	0.00313	24444	29.571	< 0.001

SE, standard error; df, degree of freedom.

Table S4. Matrix of pairwise F_{ST}(lower diagonal) and p values (upper diagonal) based on the total mMSAP and uMSAP data of the 17 populations of *Z. kawagoii* estimated using ARLEQUIN. Significance was determined by 10,000 permutations. See **Table 1** for population code.

	**AT**	**BTWS**	**EFS**	**HDD**	**JS**	**JSY**	**KTS**	**LY**	**NZ**	**RF**	**SBS**	**SL**	**SML**	**THS**	**TRK**	**WL**	**WLS**
mMSAP																	
AT		0	0	0	0	0	0	0	0	0	0.0001	0	0	0	0	0	0
BTWS	0.093		0.033	0.000	0.000	0.035	0.000	0.017	0.000	0.020	0.039	0.070	0.000	0.001	0.000	0.150	0.109
EFS	0.086	0.033		0.012	0.000	0.022	0.000	0.004	0.000	0.235	0.152	0.035	0.000	0.018	0.000	0.224	0.045
HDD	0.050	0.090	0.047		0.000	0.000	0.000	0.000	0.001	0.004	0.005	0.001	0.000	0.002	0.000	0.002	0.000
JS	0.077	0.131	0.090	0.075		0.000	0.000	0.000	0.000	0.000	0.000	0.000	0.000	0.000	0.002	0.000	0.000
JSY	0.070	0.031	0.043	0.069	0.095		0.000	0.031	0.000	0.004	0.035	0.057	0.000	0.002	0.000	0.065	0.007
KTS	0.055	0.114	0.092	0.042	0.079	0.081		0.000	0.000	0.000	0.000	0.000	0.000	0.000	0.000	0.000	0.000
LY	0.094	0.041	0.066	0.083	0.133	0.033	0.110		0.000	0.002	0.007	0.006	0.000	0.000	0.000	0.124	0.002
NZ	0.080	0.112	0.082	0.042	0.054	0.079	0.069	0.103		0.000	0.005	0.000	0.000	0.000	0.000	0.000	0.000
RF	0.069	0.040	0.007	0.054	0.102	0.057	0.077	0.076	0.088		0.134	0.006	0.000	0.010	0.000	0.109	0.070
SBS	0.056	0.030	0.013	0.054	0.072	0.030	0.072	0.051	0.053	0.014		0.018	0.000	0.025	0.000	0.193	0.015
SL	0.091	0.024	0.036	0.066	0.121	0.025	0.102	0.052	0.092	0.054	0.042		0.000	0.005	0.000	0.069	0.009
SML	0.087	0.122	0.072	0.056	0.051	0.089	0.078	0.111	0.061	0.103	0.080	0.094		0.000	0.000	0.000	0.000
THS	0.073	0.085	0.046	0.046	0.074	0.065	0.096	0.082	0.058	0.052	0.039	0.054	0.065		0.000	0.013	0.001
TRK	0.071	0.111	0.064	0.048	0.026	0.078	0.071	0.112	0.043	0.085	0.062	0.100	0.043	0.069		0.000	0.000
WL	0.072	0.013	0.008	0.054	0.103	0.024	0.081	0.021	0.084	0.019	0.010	0.025	0.080	0.054	0.073		0.114
WLS	0.148	0.016	0.030	0.134	0.188	0.062	0.178	0.090	0.170	0.025	0.047	0.058	0.182	0.121	0.160	0.020	
Average	0.080	0.062	0.046	0.052	0.069	0.042	0.058	0.047	0.041	0.039	0.041	0.033	0.023	0.019	0.015	0.061	0.023
uMSAP																	
AT		0	0	0	0	0	0	0	0	0	0	0	0	0	0	0	0
BTWS	0.265		0.121	0.001	0.000	0.107	0.000	0.059	0.000	0.285	0.107	0.168	0.000	0.019	0.000	0.329	0.084
EFS	0.251	0.031		0.008	0.000	0.047	0.000	0.024	0.000	0.231	0.278	0.052	0.007	0.090	0.000	0.227	0.029
HDD	0.271	0.207	0.156		0.000	0.000	0.000	0.000	0.000	0.001	0.002	0.000	0.000	0.001	0.000	0.000	0.000
JS	0.281	0.250	0.172	0.197		0.000	0.000	0.000	0.000	0.000	0.001	0.000	0.000	0.000	0.000	0.000	0.000
JSY	0.203	0.044	0.070	0.169	0.202		0.000	0.046	0.000	0.048	0.071	0.137	0.000	0.009	0.000	0.092	0.016
KTS	0.178	0.270	0.208	0.203	0.203	0.212		0.000	0.000	0.000	0.000	0.000	0.000	0.000	0.000	0.000	0.000
LY	0.288	0.077	0.114	0.234	0.281	0.076	0.307		0.000	0.011	0.009	0.028	0.000	0.000	0.000	0.089	0.002
NZ	0.211	0.240	0.181	0.171	0.167	0.170	0.200	0.248		0.000	0.006	0.000	0.000	0.000	0.000	0.000	0.000
RF	0.238	0.006	0.016	0.184	0.214	0.067	0.254	0.125	0.232		0.236	0.086	0.000	0.031	0.000	0.271	0.096
SBS	0.201	0.055	0.002	0.172	0.161	0.067	0.198	0.130	0.156	0.018		0.041	0.000	0.088	0.000	0.182	0.019
SL	0.285	0.026	0.072	0.230	0.292	0.029	0.298	0.106	0.270	0.070	0.086		0.000	0.004	0.000	0.161	0.046
SML	0.252	0.232	0.137	0.174	0.182	0.154	0.160	0.254	0.173	0.206	0.145	0.238		0.000	0.000	0.000	0.000
THS	0.209	0.129	0.064	0.124	0.172	0.106	0.170	0.173	0.128	0.105	0.056	0.157	0.116		0.000	0.025	0.000
TRK	0.270	0.259	0.207	0.196	0.154	0.201	0.185	0.290	0.125	0.244	0.192	0.294	0.182	0.137		0.000	0.000
WL	0.271	0.007	0.014	0.153	0.224	0.054	0.246	0.060	0.207	0.011	0.035	0.042	0.219	0.105	0.228		0.070
WLS	0.461	0.052	0.111	0.389	0.435	0.176	0.462	0.237	0.436	0.075	0.150	0.113	0.409	0.286	0.460	0.090	
Average	0.259	0.118	0.103	0.163	0.168	0.092	0.155	0.110	0.108	0.082	0.086	0.085	0.058	0.048	0.043	0.092	0.023

Table S5. F_{ST} outliers identified by BAYESCAN and DFDIST strongly associated with environmental variables. Codes below the environmental columns represent strong correlations between F_{ST} outliers and environmental variables identified using LFMM (L), Samβada (S), and rstanarm (R).

**Locus**	**BAYESCAN**	**DFDIST**	**Environmental Variable**													
	**log₁₀(PO).**	**F_{ST}**	**Aspect**	**Slope**	**BIO7**	**BIO9**	**BIO12**	**BIO19**	**CLO**	**EVI**	**LAI**	**MI**	**NDVI**	**PET**	**Soil pH**	**WS_{mean}**
mMSAP																
mX03HM_4577	4.2218	0.2518		R									R	R	R	R
mX06HM_2011	1.1753	0.1185		R			S									R
mX07HM_2135	2.0847	0.1313		R										R	R	R
mX07HM_2602	4.3979	0.1693		LR												L
mX07HM_3712	1000	0.2727	L	L	LS	L	LS			L	S		L		L	L
mX09HM_4962	2.6769	0.2121												R	R	LR
mX10HM_2924	2.4854	0.1308	LSR		LS		L			L	R	L	LSR	R	L	L
mX10HM_4449	2.6566	0.1827	LSR		LS	L	L			LS			L		L	L
mX10HM_4950	1.3389	0.1653		R							R			R	R	
mX20HM_2494	1000	0.2537	LSR		L		LS						R	R		LR
uMSAP																
uX01HM_2007	1000	0.2810		LR			R			L		L				LR
uX01HM_2132	1000	0.3360		R	L	L				L	R		LR		R	LSR
uX03HM_1635	1000	0.3189	LSR		LSR						R		R		R	
uX03HM_3598	1000	0.3546		R	L	LSR				L	R		LR		R	LSR
uX06HM_2011	1000	0.3240	LSR	R	LS	LR	LS	L	L		R	L		LS		
uX06HM_2055	2.6045	0.4264					R			S		SR				
uX06HM_3270	1000	0.3444	SR	LS	L	L	S			L		LSR	R			
uX07HM_1962	1000	0.3008		R		R	LS				R		R	L	R	R
uX07HM_2326	1000	0.4354	LSR		LSR	LSR		L		L		L		LS	R	
